# *Perapion connexum* (Schilsky, 1902) (Coleoptera, Apionidae) in Central Europe, a case of plant expansion chase

**DOI:** 10.3897/zookeys.174.2526

**Published:** 2012-03-09

**Authors:** Marek Wanat, Attila Podlussány, Karel Schön

**Affiliations:** 1 Museum of Natural History, University of Wrocław, Sienkiewicza 21, 50-335 Wrocław, Poland; 2 Hungarian Natural History Museum, Budapest, Hungary; 3 Valdštejnská 2108, 436 01 Litvínov, Czech Republic

**Keywords:** Weevils, Curculionoidea, *Perapion*, Poland, Hungary, Slovakia, Kyrgyzstan, new records, morphology, taxonomy, key, biology, *Rumex confertus*

## Abstract

*Perapion connexum* (Schilsky) is recorded for the first time from Hungary and Kyrgyzstan, and new distribution data from Ukraine and Russia are provided. Preliminary placements of this weevil in faunal checklists for Poland and Slovakia are here documented with detailed data. Its occurrence in Austria based on older evidence, is discussed. The neophytic and invasive in Central Europe sorrel *Rumex confertus* Willd. is confirmed to be its unique host plant in Poland. Morphology of the newcoming weevil is described and illustrated, and the key to all Central European species of *Perapion* is presented.

## Introduction

The title weevil species was originally described from Aulie-Ata (currently Taraz) in SE Kazakhstan and long considered as confined to Asian fauna (Schilsky 1902, Wagner 1930). After the World War II it was found on several distant localities in the European part of Russia, both in the extreme south (vicinity of Krasnodar) and well north of the 50^th^ parallel of north latitude (Bryansk, Ul’yanovsk), as well as in Eastern Ukraine (Luhansk and Kharkiv regions) and in Moldova (Korotyaev 1987, Poiras 1998). Further published records concerned Miodobory and Podolian Upland in the Western Ukraine (Mazur and Kuśka 1994, Mazur 2002). Most recently it was found in Kiev (Nazarenko 2011). At the same time the species turned out widespread in Western Siberia, in the provinces of Tomsk, Novosibirsk, Kemerovo, and in the Altai (Legalov 1998, 2002; Legalov and Opanasenko 2000, Krivets and Legalov 2002), thus covering practically the whole natural range of its main host plant, *Rumex confertus* Willd. The new sites of *Perapion connexum* presented herein are located more to the west, and partially within the boundaries of Central Europe, where the plant is an invasive neophyte, and the weevil remained completely unknown hitherto. The aims of this paper are to document its current distribution in Western Palaearctic, and to facilitate its recognition from other species of *Perapion* occurring in Central Europe.

## Material and methods

The study was based on 118 specimens collected by the authors in 2000-2008, obtained from other collectors or borrowed from several institutional collections.

Measurements were taken using a calibrated stereomicroscopic grid eyepiece. Body length excludes rostrum, but includes head; it was measured in lateral view from the anterior eye margin to the apex of the elytra. Width of head was measured across middle of eyes. Tarsal width was measured at the level of the 3^rd^ segment.

Photos of specimens were taken with a Leica M205C stereomicroscope and attached JVC KYF75 digital camera, and processed using the AutoMontage Pro and Adobe Photoshop CS2 software programmes.

The abbreviations used are as follows: MW, AP, KS – authors’ respective acronyms, HG – G. Hegyessy, KMS – Kazinczy Museum, Sátoraljaújhely, nr. – near. Unless elsewhere stated, voucher specimens are in the collector’s collections.

## Taxonomic treatment

### Perapion connexum

(Schilsky, 1902)

http://species-id.net/wiki/Perapion_connexum

Apion connexum Schilsky, 1902: 28.
Apion arcuatum Bajtenov, 1977: 15. Syn. by Legalov (1998).

#### Diagnosis.

*Perapion connexum* is of the same size and at first glance very similar to the common in Europe *Perapion curtirostre*, from which it differs in a black tone of body integument (evidently grey in *curtirostre*), almost cylindrical and distinctly curved rostrum (thickened in basal half and nearly straight in *curtirostre*, as in Figs 5, 6, 9, 10), narrower subconical head, puncturation of vertex rugose and indefinite, smaller and not elongate scutellum (scutellar shield), slenderer tarsi and in male metatarsi devoid of ventral spines. It strongly resembles *Aizobius sedi* in the colour of integument, but the latter species has different frons sculpture, with well defined punctures and long median fovea, pronotum distinctly rounded at sides, and a ventrally spined basal segment on all male tarsi. See the key to species of *Perapion* occurring in Central Europe given below.

**Figures 1–5.**
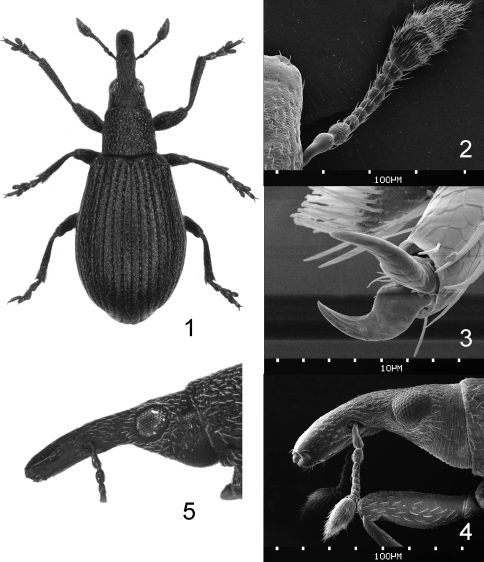
**1–4** *Perapion connexum* (Schilsky), female **1** total view **2** antenna **3** tarsal claws **4** head with rostrum, lateral view **5** *Perapion curtirostre* (Gyllenhal), female head with rostrum, lateral view. **2–4** SEM photos.

**Figures 6–11.**
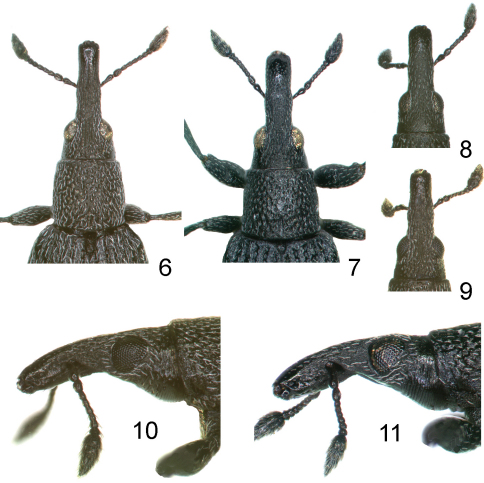
**6, 7** female anterior half of body, dorsal view **6** *Perapion curtirostre* (Gyllenhal) **7** *Perapion connexum* (Schilsky) **8, 9** male head with rostrum, dorsal view **8** *Perapion connexum* **9** *Perapion curtirostre* **10, 11** same in lateral view **10** *Perapion curtirostre*, **11** *Perapion connexum*.

#### Morphology.

Body length 2.0–2.3 mm.

*Integument and vestiture.* clearly black with slight “oily” glint (Fig. 1). Body covered with sparse and extremely fine white-semitransparent hair-like scales, on pronotum as long as diameter of the largest punctures, on elytral disc not longer than half interval’s width and unordered on intervals, not aggregated in any part of elytra, slightly denser on mesothoracic epimera and anepisterna, along metanepisterna condensed to form a thin white line. Entire body surface with dense microreticulation, scale-like and rough on head and the basal half of rostrum.

*Rostrum* in dorsal view subcylindrical with obtuse widening at antennal insertion, obscuredly punctured throughout, except distal third completely mat.

*Head* narrow, subconical, nearly as long as wide, about 1.5× narrower than pronotum (Figs 7, 8); eyes gently convex; frons slightly depressed in middle, with a few indictinct strigae partly obscured by dense microsculpture; puncturation on vertex lacking or indefinite, rarely with few punctures much smaller than on pronotal disc; head ventrally between eyes evenly scale-like microsculptured, without irregular asperities.

*Antennae* short and thin, with large club nearly as long as six distal funicular segments combined, 2.10–2.25× as long as wide, having fused segments with their circular rims incomplete (Fig. 2); pedicel 1.4–1.6× longer than wide, twice as long as next segment, segments 2, 3 minute and weakly elongate, segments 4, 5 isodiametric, 6 sligthtly, and 7 markedly transverse.

*Pronotum* small, slightly shorter than wide, with weakly rounded sides, at base 1.1–1.2× as wide as at apex, coarsely punctured, the punctures usually of 3–4 combined ommatidia size, with flat, heavily and somewhat roughly microreticulate interspaces; prescutellar fovea not wider than single puncture, as long as 3–4 neighbouring punctures combined.

*Scutellar shield* small, isodiametric (Fig. 7).

*Elytra* widest clearly behind mid-length, 1.6–1.7× longer than wide, 3.4–3.8× as long as pronotum, with deeply impressed catenulate-punctate striae, on elytral disc half as wide as intervals; intervals flat, barely punctate; specialised setae single on 7^th^ and 9^th^ interval.

*Wing* without radial window.

*Ventrites*. Metaventrite and abdominal ventrites I, II microreticulate and evently punctate, shiny, the punctures much smaller than on pronotal disc, well over a diameter apart from each other; abdominal ventrites III–V with strong, scale-like microsculpture.

*Legs* slender; profemur 0.80–0.85× as thick as rostrum; protibia widening from base to apex, with obsolescent apical tuft of setae; tarsi slender, protarsus 3.15–3.40× as long as wide; claws untoothed, thickened basally (Fig. 3).

Male. Rostrum slightly shorter than pronotum, 2.20–2.35× longer than wide, in profile almost straight and somewhat wedge-like, distinctly narrowing apicad in distal half (Fig. 11). Antennal insertion at basal 0.38–0.42 of rostrum. Abdominal ventrite V very broadly rounded apically. Metatarsus unarmed. Pygidium half exposed, with very broad complete transverse sulcus. Terminalia only slightly different from those of *Perapion curtirostre*, mainly in more elongate tegminal plate and aedeagus. Sternite VIII broad, with very short and indistinct lobes. Sternite IX with slightly asymmetrical fork half as long as apodeme. Tegmen with phallobase as long as apodeme; tegminal plate fused, short, devoid of macrochaetae, with broadly and very deeply emarginate prostegium. Aedeagus short and flattened, with pedon about 4.5× as long as wide, membranous tectum and free apophyses less than 0.2× as long as pedon; endophallus finely and more or less evenly microspinose.

Female. Rostrum 1.00–1.15× as long as pronotum, 2.60–2.75× longer than wide, in profile distinctly curved and equally high along its length (Fig. 4). Antennal insertion at basal 0.35–0.39 of rostrum. Abdominal ventrite V narrowly rounded apically. Tergite VIII broad and strongly transverse, uniformly slerotized. Sternite VIII with large and broad basal arms. Gonocoxites less than 2.5× longer than wide, without median string of sclerotisation; styli slightly elongate, shortly setose apically.

#### Material examined. 

Poland (E): Stare Stulno (51.3714°N, 23.6628°E), 1 VIII 2000, 4 exs, 2 VIII 2000, 10 exs, 5 VIII 2000, 10 exs, 7 VI 2001, 1 ex., 31 VII 2001, 16 exs; Rudka nr. Wola Uhruska (51.2761°N, 23.6694°E), 15 VII 2002, 1 ex.; Wołczyny (51.4392°N, 23.6656°E), 6 VII 2002, 2 exs; Orchówek-Obłonie (51.5291°N, 23.5950°E), 7 VII 2002, 2 exs; Sobibór (51.4680°N, 23.6599°E), 6 VII 2002, 13 exs; Kosyń (51.3903°N, 23.5750°E), 12 VII 2002, 7 exs; Hniszów (51.2646°N, 23.7119°E), 15 VII 2002, 2 exs – all leg. et coll. MW.

Ukraine (W): Podolia: Zvenihorod at Dniester riv., 48.5500°N, 26.2833°E, 25 VI 1996, 2 exs; Kamyanets Podilskiy, 48.6667°N, 26.5667°E, 26 VI 1996, 2 exs – all leg. et coll. MW;

Hungary: Borsod-Abaúj-Zemplén county: Füzér, Hosszú-rét (48.5644°N, 21.4324°E), 21.VII.2005, 3 ex., leg. Hegyessy G & S; Alsószuha, Hideg-kút-völgy (48.3586°N, 20.5144°E), 17 VI 2003, 5 exs, leg. HG – coll. AP (2 ex) and KMS (3 exs); Zalkod, Erkecse (48.1818°N, 21.4541°E), 10.VII.1993, 1 ex, leg. HG – coll. KMS; Szalonna, Köszvényeskút (48.4612°N, 20.7086°E), 10.V.2007, 1 ex, leg. HG – coll. KMS; Tornaszentandrás: Mile-völgy (48.5066°N, 20.7853°E), 10.V.2007, 1 ex, leg. HG – coll. KMS; Mád, Becsek (48.1826°N, 21.3056°E), 10.IV.2008, 1 ex, leg. HG & AP – coll. AP; Taktaszada: Ökör-mező (48.1122°N, 21.1504°E), 11.VI.2008, 1 ex, leg. HG – coll. KMS.

Slovakia (S, E): Železné env., Tornaľa - Starňa (48.4167°N, 20.4000°E), 26 V 2006, 1 ♂, leg. et coll. T. Kopecký; Zemplínské Kopčany (48.5833°N, 21.8833°E), 14 VI 2000, 1 ♂ 1 ♀, leg. P. Boža – coll. S. Benedikt, 20 V 2002, 7 ♂♂ 7 ♀♀, leg. M. Mantič – coll. M. Mantič & KS; Turňa nad Bodvou (48.6000°N, 20.8667°E), 9 VI 2001, 1 ♂, leg. R. Fornůsek – coll. S. Benedikt.

Russia: Kursk, 1 ex.; Orel [Oryol], 3 exs; Nikitskoe near Voronezh, 1 ex. – all coll. F. Schubert (in Naturhistorisches Museum Wien). W Siberia: Novosibirsk Area, Kochenevo distr., 43 km WNW of Kochenevo, Sektinskoye Lake, 27.05.1998, leg. R. Dudko & A. Legalov, det. A. Legalov, 6 exs – coll. KS (2 ♂♂ 2 ♀♀) and M. Koštál (1 ♂ 1 ♀). Rostov reg.: Krasny Sulin distr.: Donleskhoz env. (47.8627°N, 40.2405°E), 12 VI 2004, 1 ex., leg. D. Kasatkin – coll. MW.

Kyrgyzstan: Chüy province: Ala-Archa valley (42.6000°N, 74.4833°E), ca. 30 km S of Bishkek, above 1300 m alt., 4 VI 2003, 3 exs., leg. R. Królik – coll. MW.

#### Distribution. 

Austria?, Hungary*, Kazakhstan, Kyrgyzstan*, Moldova, Poland (E), Russia (Central and South European Territory, Western Siberia), Slovakia (S and E), Ukraine, Uzbekistan (first records herein marked with asterisk).

#### Biology.

Korotyaev (1987) collected this weevil from broad-leaved sorrel species. The senior author (MW) collected it in Ukraine by general sweeping of wet meadows in the Dniester valley, where an unidentified broad-leaved sorrel was abundant. Poiras (1998) identified the host plant as *Rumex confertus* Willd. and, indeed, in Poland the weevil was collected exclusively from this sorrel species. In the Udmurt Republic Dedyukhin (2009) confirms the same host plant, but he collected adults also from the sorrels resembling *Rumex crispus* L. The life cycle of *Perapion connexum* remains unknown, but the adults were in Poland mostly beaten in summer from mature infruitescences, which may indicate larval feeding on developing seeds or eventually in fruit petioles, rather than in thick main stem or leaf petioles. In Poland teneral beetles were observed since mid-July.

#### Comments.

Korotyaev (1987) reported a specimen from the collection of ZIN labelled “Austria”, which was then approximately 800 km distant from the westernmost known locality in Moldova. This outstanding record was ignored by the authors of subsequent Centraleuropean weevil catalogues (Lucht 1987, Böhme 2005, Alonso-Zarazaga 2011), but in the light of our current findings and proximity of current Slovak and Hungarian localities, this opinion should be verified and the occurrence of *Perapion connexum* in Austria should be considered as likely, though obviously requiring confirmation with new data. Unfortunately, the information on distribution of its host plant in Austria is poor and equivocal. It was missing from the first two editions of Austrian Excursionsflora by Fritsch (1897, 1909), but it was noticed from Austria since at least mid-20^th^ c. (Tutin et al. 1964). Then Jalas and Suominen (1979) did not justify Austrian records of this sorrel, and they were consequently removed from the revised editions of Flora Europaea. Most recently the occurrence of *Rumex confertus* in Austria has been confirmed in the departments of Wien, Niederösterreich, Steiermark and Kärnten (Fischer et al. 2008), but the history of its invasion(s) remains unclear.

The occurrence of *Perapion connexum* in Poland, as based on the abovementioned data, was earlier generally announced by Wanat and Mokrzycki (2005), and further confirmed by Gosik (2006). Analogously, the weevil has been just placed on the list in Slovakia (Benedikt et al. 2010). The range of this weevil in Poland seems still strictly limited to the southern section of the Bug River Valley, which constitutes there the country border between Poland and Ukraine, but one of the listed localities (Kosyń) is situated ca. 18 km “inland” West of the river. Along the Bug River Valley the southernmost site is Gródek near Hrubieszów (Gosik 2006) (lat/long approximately 50.79°N, 23.96°E), while the remaining seven sites are situated between Hniszów and Orchówek, which is the northermost locality of this species in Poland (51.5291°N, 23.5950°E). Searching for the weevil in 2002-2003 in similar sites rich of the host plant but laying North along the Bug valley, i.e. in Parośla nr. Sławatycze (51.8099°N, 23.6206°E), Mielnik (52.3328°N, 23.0225°E) and Kózki nr. Siemiatycze (52.3605°N, 22.8660°E), brought negative results. Nevertheless, in Russia the weevil was found up to 54.5°N in Ul’yanovsk (Korotyaev 1987) and even 57°N in the Udmurt Republic (Dedyukhin 2009) and the northernmost Siberian sites (Legalov 2002), despite of continental climate. Thus the Lower Bug Valley seems to be the most obvious natural area for further spreading of *Perapion connexum* in Poland and presently limited range of the weevil there may indicate a stage of current invasion.

*Rumex confertus* is an invasive plant in Europe, and its natural range ends probably close to Southeastern Poland, in Southern Slovakia and Hungary (Rechinger and Schreiber 1957, Tutin et al. 1964, Jalas and Suominen 1979, Dostál 1989, Jehlík et al. 2001). However, although it is known from the Bug River Valley in Poland since 1873 (Eichler and Łapczyński 1892), its autochtonous status in Poland is doubtful. According to Trzcińska-Tacik (1963) and Tacik (1992), who studied distribution of this sorrel species in most detail, its natural range North of the Carpathians rather ends in Western Ukraine. Its spreading to the West of Poland started probably since 1950 (Tokarska-Guzik 2005) and currently it appears a common plant in Poland east of the Vistula river, reaching even the Baltic coast to the North, and it has many diffused localities also in the Western Poland (Trzcińska-Tacik 1963, Zając and Zając 2001, Stosik 2006). It extends its range widely also to the North, being probably introduced to Skandinavia with the Soviet army transports since the very early 20^th^ century in Finland, and about mid 20^th^ century in Norway and Sweden (Snogerup 2000). It is now widespread also in Baltic countries and treated as invasive plant in Lithuania (Gudžinskas 1999). The Southern stream of its invasion to Central Europe seems less active. The plant is still very rare in Czech Rep. with just a few isolated and ephemeral localities (Jehlík et al. 2001) and, as stated above, it has quite similar status in Austria.

**Figure 12.**
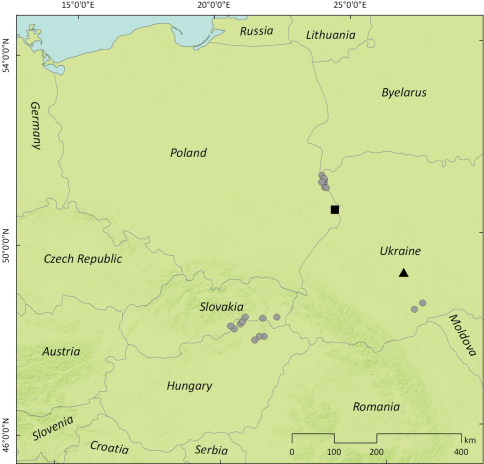
Westernmost recent localities of *Perapion connexum* (Schilsky) in Central Europe mentioned in the text (circles – new records; black square – record by Gosik (2006) in Gródek nr. Hrubieszów, Poland; black triangle – record by Mazur and Kuśka (1994) in Vikno, Ukraine).

Following current distribution of the host plant, further expansion of *Perapion connexum* in Central Europe from the sites showed in Fig. 12 seems very likely especially through the territory of Poland, and it could be monitored quite easily by summer sweeping of mature inflorescences of *Rumex confertus*. The same method should be applied on stabilized localities of *Rumex confertus* in Austria to record its occurrence and expansion.

## Key to Central European species of Perapion^†^:

The key includes also *Aizobius sedi* (Germar, 1818), a related and morphologically similar species.

**Table:** 

1	Elytra with distinct metallic blue or green shine	2
–	Elytra black, concolorous with rest of body	5
2	Body longer than 2.5 mm. Scutellar shield elongate, subrectangular. Elytra 1.5–1.7× longer than wide. Male basal segment of metatarsus with a ventral spine	3
–	Body shorter than 2.5 mm. Scutellar shield isodiametric, rather triangular. Elytra nearly always 1.4-1.5× longer than wide. Tarsi without ventral spines	4
3	Abdominal ventrites 1-2 entire coarsely punctate, punctures nearly as large as those on pronotal disc. Pronotum 1.4-1.7× wider than head across eyes. Rostrum slightly curved, in male at least 2.5× longer than its basal width, in female longer than pronotum and more or less cylindrical	*Perapion violaceum* (Kirby)
–	Abdominal ventrites 1-2 sparsely and finely punctate, punctures several times smaller than those on pronotal disc. Pronotum at most 1.2–1.4× wider than head across eyes. Rostrum shorter, straight, in male tapering from base to apex and less than 2.5× longer than its basal width, in female as long as or shorter than pronotum, narrowing apicad from antennal insertion	*Perapion hydrolapathi* (Marsham)
4	Genae coarsely punctured and finely strigose. Punctures on head dorsum and pronotum mostly slightly elongate	*Perapion affine* (Kirby)
–	Genae largely impunctate, only transversely strigose. Punctures on head dorsum and pronotal disc round	*Perapion marchicum* (Herbst)
5	Frons with evident median sulcus, though not wider nor deeper than neighbouring punctures. Pronotum with markedly rouded sides and very thick walls, its disc distinctly convex. In male all tarsi ventrally spinose. On Crassulaceae (*Sedum* spp.)	[*Aizobius sedi* (Germar)]
–	Frons finely punctate or strigose. Pronotum with weakly rounded to nearly straight sides and thinner walls, its disc barely convex. At most male metatarsi with ventral spines. On Polygonaceae (*Rumex* spp., *Polygonum* spp.)	6
6	Body vestiture distinct, composed of cream-yellowish hair-like scales, on elytra forming a condensed patch on the outermost interval along metathoracic ventrite. Rostrum cylindrical, thinner than profemur. On *Polygonum aviculare* L.	*Perapion lemoroi* (Ch. Brisout)
–	Body vestiture less distinct, the scales finer, evenly confused on elytra. Rostrum at base as thick as or thicker than profemur	7
7	Head and basal half of rostrum with indistinct puncturation obscured by very dense rough scale-like microreticulation. Rostrum in female distinctly curved, in male somewhat wedge-like due to prominent septum between antennal insertions (Figs 4, 11). Scutellar shield isodiametric, flat (Fig. 7). Body vestiture very fine, beetle appears evidently black. Male tarsi unarmed. On *Rumex confertus* Willd	*Perapion connexum* (Schilsky)
–	Head and basal half of rostrum clearly punctured, the punctures dense and on vertex nearly as large as those on pronotal disc. Rostrum straight to slightly arched, in lateral view equally high throughout (Figs 5, 10). Scutellar shield elongate, furrowed (Fig. 6). Body vestiture more distinct, altogether with integument microsculpture giving beetle a greyish colouration. In male basal segment of metatarsus with small ventral spine	8
8	Body larger, 2.2-3.0 mm long. Rostrum longer, in male ca. 3×, in female ca. 3.5× longer than its width at antennal insertion, in female subequally wide at narrowest points before and behind antennal insertion. On *Rumex acetosa* L.	*Perapion oblongum* (Gyllenhal)^‡^
–	Body smaller, 1.5-2.6 mm long. Rostrum in male ca. 2.5×, in female ca. 3.0× longer than its width at antennal insertion, in female at narrowest point basad of antennal insertion clearly wider than at narrowest point of apical half of rostrum (Fig. 6). On a wide range of *Rumex* spp., occasionally on *Polygonum bistorta* L.	*Perapion curtirostre* (Germar)

† Generic definition after Alonso-Zarazaga (1990)

‡ This species was synonymised with *Perapion curtirostre* by Legalov (2001), which was commented by Wanat and Mokrzycki (2005), refused by Alonso-Zarazaga (2011), and is not agreed upon by the authors of this paper.

## Supplementary Material

XML Treatment for Perapion connexum

## References

[B1] Alonso-Zarazaga MA (1990) Revision of the supraspecific taxa in the Palaearctic Apionidae Schoenherr, 1823 (Coleoptera, Curculionoidea). 2. Subfamily Apioninae Schoenherr, 1823: introduction, keys and descriptions. Graellsia 46: 19-156

[B2] Alonso-Zarazaga MA (2011) Apionidae. In: Löbl I, Smetana A (Eds) Catalogue of Palaearctic Coleoptera. Vol. 7: Curculionoidea I. Apollo Books, Stenstrup, 373 pp.

[B3] Bajtenov MS (1977) Materialy k palearkticheskim vidam roda *Apion* Herbst (Col. Curculionidae). Izvestia Akademii Nauk Kazakhskoy SSR, Seriya Biologicheskaya 4: 13-18

[B4] Benedikt S Borovec R Fremuth J Krátký J Schön K Skuhrovec J Trýzna M (2010). Annotated checklist of weevils (Coleoptera: Curculionoidea excepting Scolytinae and Platypodinae) of the Czech Republic and Slovakia. Part 1. Systematics, faunistics, history of research on weevils in the Czech Republic and Slovakia, structure outline, checklist. Comments on Anthribidae, Rhynchitidae, Attelabidae, Nanophyidae, Brachyceridae, Dryophthoridae, Erirhinidae and Curculionidae: Curculioninae, Bagoinae, Baridinae, Ceutorhynchinae, Conoderinae, Hyperinae. Klapalekiana 46 (Supplement): 1–363.

[B5] Böhme J (2005) Die Käfer Mitteleuropas. Band K. Katalog (Faunistische Übersicht). 2. Auflage (begründet von W. H. Lucht). Elsevier GmbH, Spektrum AV, xii + 515 pp.

[B6] Dedyukhin SV (2009) Materialy k faune dolgonosikoobraznykh zhestkokrylykh (Coleoptera, Curculionoidea) nationalnogo parka “Nechkinskiy”. Vestnik Udmurtskogo Universiteta, Seriya Biologiya i Nauki o Zemlye 2: 34-48

[B7] Dostál J (1989) Nová květena ČSSR. Akademia, Praha, 1548 pp.

[B8] Eichler B Łapczyński K (1892) Korespondencya do Wrzechświata. Wrzechświat 11: 814-815

[B9] Fritsch K (1897) Excursionsflora für Österreich (mit Ausschluss von Galizien, Bukovina und Dalmatien). Karl Gerold’s Sohn, Wien, LXXII + 664 pp.

[B10] Fritsch K (1909) Excursionsflora für Österreich (mit Ausschluss von Galizien, Bukovina und Dalmatien). Edition 2. Karl Gerold’s Sohn, Wien, LXXX + 725 pp.

[B11] Gosik R (2006) Weevils (Curculionoidea) of the middle part of the Bug River Valley. Annales Universitatis Mariae Curie-Sklodowska, Sectio C 61: 7-69

[B12] Gudžinskas Z (1999)Botanica Lithuanica 5: 313-326

[B13] Jalas J Suominen J (Eds) (1979) Atlas florae europaeae. Vol. 4, Polygonaceae. Committee for Mapping the Flora of Europe and Societas Biologica Fennica Vanamo, Helsinki, 71 pp. + 94 maps.

[B14] Jehlík V Sádlo J Dostálek J Jarolímova V Klimeš L (2001) Chorology and ecology of *Rumex confertus* Willd. in the Czech Republic. Botanica Lithuanica 7: 235-244

[B15] Korotyaev BA (1987) Materialy k poznaniyu zhukov nadsemeystva Curculionoidea (Coleoptera) fauny SSSR i sopredelnykh stran. Trudy Zoologicheskogo Instituta Akademii Nauk SSSR 170: 122-163

[B16] Krivets SA Legalov AA (2002) Obzor zhukov nadsem. Curculionoidea (Coleoptera) fauny Kemerovskoy Oblasti. Entomologicheskoye Obozreniye 81: 817-833

[B17] Legalov AA (1998) Fauna dolgonosikoobraznykh zhukov semeystv Nemonychidae, Urodonidae, Anthribidae, Attelabidae, Apionidae i Dryophthoridae Zapadnoy Sibirii. Bespozvonochnye zhivotnye Yuzhnogo Zaural’a i sopredelnykh territoriy, Kurgan, 216–221.

[B18] Legalov AA (2001) Novyi sinonim v rode *Perapion* (Coleoptera, Apionidae). Vestnik Zoologii 35: 78.

[B19] Legalov AA (2002) Spisok zhukov semeystv Nemonychidae, Urodontidae, Rhynchitidae, Attelabidae i Brentidae (Coleoptera, Curculionoidea) Aziatskoy Rossii. Zhivotnyi Mir Daln’ego Vostoka, Blagoveshtschensk, 4: 105-116

[B20] Legalov AA Opanasenko FI (2000) Obzor zhukov nadsem. Curculionoidea (Coleoptera) fauny Novosibirskoy Oblasti. Entomologicheskoye Obozreniye 79: 375-395

[B21] Lucht W (1987) Die Käfer Mitteleuropas. Katalog. Goecke & Evers, Krefeld, 342 pp.

[B22] Mazur M (2002) The distribution and ecology of weevils (Coleoptera: Nemonychidae, Attelabidae, Apionidae, Curculionidae) in western Ukraine. Acta Zoologica Cracoviensia 45: 213-244

[B23] Mazur M Kuśka A (1994) Chrząszcze Miodoborów (Zachodnia Ukraina). I. Ryjkowce (Coleoptera: Attelabidae, Apionidae, Curculionidae) – wyniki ekspedycji w latach 1993–1994. Polskie Pismo Entomologiczne 63: 277-310

[B24] Nazarenko VYu (2011) Novaya nachodka zhuka-apiona *Perapion connexum* (Coleoptera, Apionidae) v Kievie. Vestnik Zoologii 45(5): 446.

[B25] Poiras AA (1998) Catalogue of the weevils and their host plants in the Republic of Moldova. Pensoft, Sofia - Moscow, 156 pp.

[B26] Rechinger KH Schreiber A (1957) Polygonaceae, pp. 352–436. In: Gustav Hegi Illustierte Flora von Mitteleuropa. Vol. 3(1). Carl Hanser Verlag, München, viii + 452 pp.

[B27] Schilsky J (1902) Die Käfer Europa’s. Nach der Natur beschrieben von Dr. H. C. Küster und Dr. G. Kraatz, Nürnberg, Heft 39: I-IV + 100 nrs.

[B28] Snogerup S (2000) *Rumex confertus* Willd. In: Jonsell B Karlsson T (Eds). Flora Nordica.Vol. 1. Lycopodiaceae-Polygonaceae. Bergius Foundation, Royal Swedish Academy of Sciences, Stockholm: 300-302

[B29] Stosik T (2006) Ekspansja *Rumex confertus* Willd. w kontekście jego biologii. Prace Komisji Nauk Rolniczych i Biologicznych Bydgoskiego Towarzystwa Naukowego, Seria B 59: 71-81

[B30] Tacik T (1992) *Rumex* L. Szczaw. In: Jasiewicz A (Ed.). Flora Polski.Rośliny naczyniowe 3. Instytut Botaniki im. W. Szafera, Polska Akademia Nauk, Kraków: 90-102

[B31] Tokarska-Guzik B (2005) The Establishment and Spread of Alien Plant Species (Kenophytes) in the Flora of Poland. Prace Naukowe Uniwersytetu Śląskiego w Katowicach, 1–192.

[B32] Trzcińska-Tacik H (1963) Studies on the distribution of synanthropic plants. 2. *Rumex confertus* Willd. in Poland. Fragmenta Floristica et Geobotanica 9: 73-84

[B33] Tutin TG Heywood VH Burges NA Valentine DH Walters SM Webb DA (1964) Flora Europaea. Vol. 1. Lycopodiaceae to Platanaceae. Cambridge University Press, xxxii + 464 pp, 5 maps.

[B34] Wagner H (1930) Apioninae. In: Winkler A, Catalogus Coleopterorum Regionis Palaearcticae. Pars 11. A Winkler, Wien, 1265–1392.

[B35] Wanat M Mokrzycki T (2005) A new checklist of the weevils of Poland (Coleoptera: Curculionoidea). Genus 16: 69-117

[B36] Zając A Zając M (Eds) (2001) Distribution Atlas of Vascular Plants in Poland. Laboratory of Computer Chorology, Institute of Botany Jagiellonian University, and Fundation of Jagiellonian University, Cracow, xii + 716 pp.

